# Optimized Automated Workflow for BioID Improves Reproducibility and Identification of Protein–Protein Interactions

**DOI:** 10.1021/acs.jproteome.4c00308

**Published:** 2024-09-04

**Authors:** Emilio Cirri, Hannah Knaudt, Domenico Di Fraia, Nadine Pömpner, Norman Rahnis, Ivonne Heinze, Alessandro Ori, Therese Dau

**Affiliations:** Leibniz Institute on Aging—Fritz Lipmann Institute (FLI), 07745 Jena, Germany

**Keywords:** BioID, proximity labeling, mass spectrometry, automation, high throughput

## Abstract

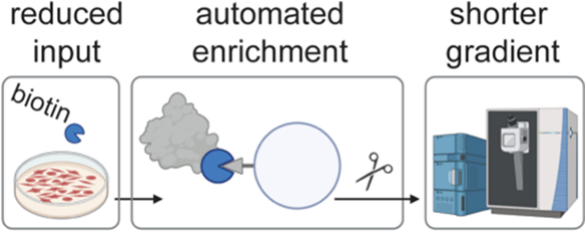

Proximity-dependent biotinylation is an important method to study protein–protein interactions in cells, for which an expanding number of applications has been proposed. The laborious and time-consuming sample processing has limited project sizes so far. Here, we introduce an automated workflow on a liquid handler to process up to 96 samples at a time. The automation not only allows higher sample numbers to be processed in parallel but also improves reproducibility and lowers the minimal sample input. Furthermore, we combined automated sample processing with shorter liquid chromatography gradients and data-independent acquisition to increase the analysis throughput and enable reproducible protein quantitation across a large number of samples. We successfully applied this workflow to optimize the detection of proteasome substrates by proximity-dependent labeling.

## Introduction

Proximity-dependent biotinylation is a well-established method to study protein–protein interactions in cells^1−3^ and it is widely regarded as a complementary method to affinity purification-based methods.^4^ Here, a promiscuous biotin ligase is tagged to the target protein. Every protein in its close vicinity will be biotinylated and can be enriched via a subsequent pulldown with streptavidin or related affinity reagents. Since the biotinylation reaction takes place in the cell, not only stable protein–protein interactions, such as protein complexes,^5^ can be detected but also more transient interactions, e.g., enzyme–substrate interactions,^6^ impact of posttranslational modifications,^7,8^ and compartmentalization,^2,9−11^ can be monitored with this type of approach.

The strong affinity of biotin to streptavidin (*K*_d_ = ∼1–10 × 10^–15^) allows for very efficient and stringent enrichment of labeled proteins.^12^ However, in turn, the elution of biotinylated proteins from streptavidin beads can be inefficient. Instead of breaking the biotin–streptavidin interaction, most current protocols use on-bead enzymatic digestion (typically with trypsin) to recover peptides derived from the captured biotinylated proteins. This strategy allows for the identification of both biotinylated proteins and their interactors. However, they suffer from two limitations. First, streptavidin can also be digested by trypsin, leading to strong contamination of highly abundant streptavidin-derived peptides. Second, the fraction of biotinylated peptides recovered is typically low because they remain bound to streptavidin after digestion. The direct identification of biotinylated peptides is desirable because it enhances the confidence of the candidate protein detection and it provides structural information for direct protein–protein interactions.^13^ Therefore, different strategies have been developed to improve the detection of biotinylation sites using specific antibodies,^13,14^ modified streptavidin, (cleavable) biotin versions,^15−19^ or enrichment of specifically biotinylated peptides.^20^ Alternatively, the elution of biotinylated peptides from streptavidin can also be achieved by applying denaturing elution buffers featuring detergents,^21^ low pH,^22^ solvents,^23^ or a combination of those,^5^ after the on-bead digestion. An ideal workflow should enable the detection of interacting proteins by coenrichment as well as the identification of biotinylated peptides to pinpoint more proximal (direct) interactions.

**Figure 1:**
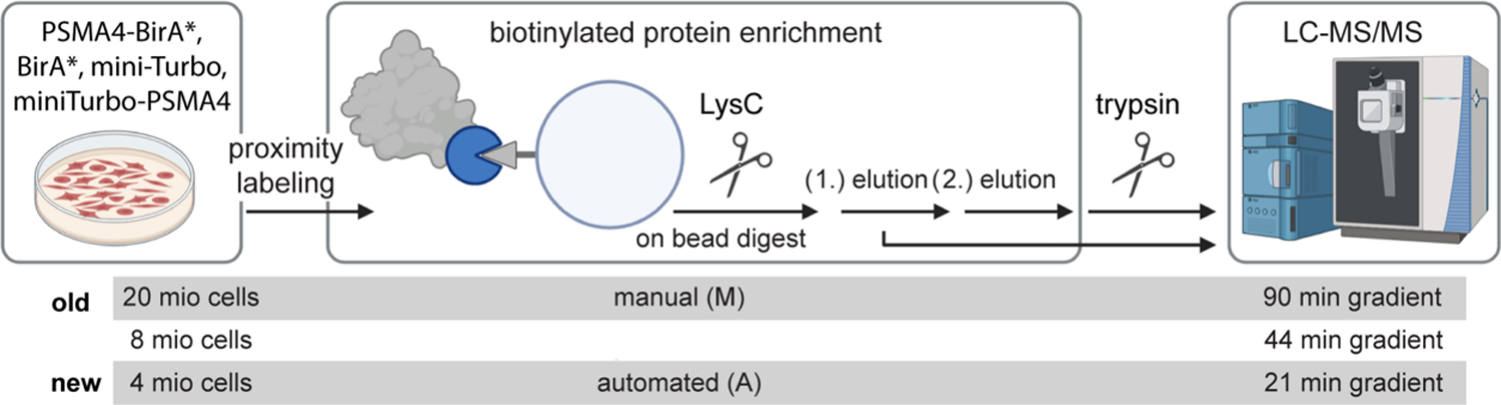
Overview of the BioID workflows compared in this study. Depicted is a schematic of the BioID workflow, showing the different parameters tested, such as cell input, enrichment procedure, and length of gradients used for mass spectrometry analysis (LC-MS/MS). HEK293T cells were grown to the desired number, and then fusion protein expression was induced with 1 μg/μL tetracycline for 4 days. For cells expressing PSMA4-BirA* or BirA*, biotin was added 24 h before harvesting, while for cells expressing PSMA4-mTurbo, mTurbo-PSMD3, or mTurbo, biotin was added 2 h before harvesting. Replicates obtained from cells at different passages were processed using manual streptavidin enrichment or with a modified version of the Agilent Bravo AssayMap On-Cartridge protocol. For both protocols, streptavidin was acetylated prior to sample loading, and proteins were on-bead digested with LysC (overnight 37 °C for manual protocol, 1 h 45 °C for automated protocol). The resulting peptides were retrieved in two elution steps: (1) Using 50 mM AmBic (first elution) followed by (2) 10% TFA in ACN (acidic second elution). Peptides were further digested off-beads using trypsin and then measured by label-free data-independent acquisition (DIA) mass spectrometry using different liquid chromatography (LC) gradients. The figure was created with Biorender.

Here, we present an automated implementation of a workflow that enables the processing of 96 samples in parallel and reduces the mass spectrometry analysis time by employing shorter chromatographic gradients (Figure 1). Importantly, our workflow yields more consistent protein quantification across replicates, enables more robust detection of biotinylated peptides, and provides a better signal-to-noise ratio thanks to a reduced unspecific binding. Finally, the higher efficiency of the automated workflow enables the input material to be reduced compared to its manual version.

## Experimental Section

### Generation of Stable Fusion Protein Cell Lines

As described by Bartolome et al.,^24^ a cell-line-expressing mTurbo-PSMD3 was generated using FlpIn T-REx 293 cells (Thermo Fisher Scientific, R78007). The parental cell line was maintained in the presence of zeocin (100 μg/mL, Thermo Fisher Scientific) and blasticidin (15 μg/mL, Thermo Fisher Scientific). After transfection, cells expressing the constructs were selected using blasticidin (15 μg/mL) and hygromycin B (100 μg/mL, Thermo Fisher Scientific). PSMA4-BirA, BirA*-, PSMA4-mTurbo-, or mTurbo-expressing cell lines have been described by Bartolome et al.^24^ All cell lines were grown at 37 °C, 5% CO_2_, and 95% humidity in Dulbecco’s modified Eagle’s medium (DMEM, Sigma-Aldrich) with high glucose (4.5 g/L), supplemented with 10% (v/v) heat-inactivated fetal bovine serum (Thermo Fisher Scientific) and 2 mM l-glutamine (Sigma-Aldrich).

### Expression of Fusion Proteins

Fusion protein expression was induced with 1 μg/μL tetracycline for 4 days. For cells expressing PSMA4-BirA* or BirA*, biotin (final concentration: 50 μM, Sigma-Aldrich) was added 24 h before harvesting. For cells expressing PSMA4-mTurbo, mTurbo-PSMD3, or mTurbo, biotin (50 μM) was added 2 h before harvesting. The proteasome inhibitor MG132 (Sigma-Aldrich) was added to a final concentration of 20 μM 4 h before harvesting. The cells were harvested with 0.05% trypsin (Thermo Fisher Scientific) and washed with phosphate-buffered saline (PBS) 3 times. Cell pellets were frozen for further usage.

### Enrichment and Digest of Biotinylated Proteins on AssayMap Bravo

For each replicate, 4, 8, or 20 Mio cells (corresponding to approximately 0.4, 0.8, or 2 mg protein) were resuspended in 250, 500 μL, or 1 mL lysis buffer (50 mM Tris pH 7.5; 150 mM NaCl; 1 mM EDTA; 1 mM EGTA; 1% (v/v) Triton 0; 10 μg/mL aprotinin (Carl Roth); 5 μg/mL leupeptin (Carl Roth); 250 U turbonuclease (MoBiTec GmbH); 0.1% (w/v) SDS), respectively, and incubated for 1 h at 4 °C. For each step, a modified version of the preset On-Cartridge protocol for Agilent Bravo AssayMap was used, and all parameters were kept constant regardless of the initial input. For acetylation of the streptavidin cartridges and subsequent loading of the lysates, the protocol was modified as follows: Cartridges were equilibrated with 200 μL of PBS (10 μL/min). For each replicate, 50 μL of 10 mM sulfo-NHS acetate was used. The reaction was set to 6 μL volume at 25 °C for 30 min. Reaction Chase was 100 μL with a flow rate of 10 μL/min. Prior to loading, cartridges were equilibrated using Internal Cartridge Wash 1, with 200 μL of lysis buffer (20 μL/min). Before and after equilibration, a Cup Wash was used with default settings. Samples were loaded at a speed of 10 μL/min. Before LysC digestion, cartridges were washed once with 200 μL of lysis buffer and 2 times with 250 μL of 50 mM ammonium bicarbonate (AmBic) (10 μL/min). For each digest, 0.5 μg of LysC (Cell Signaling) was added to 30 μL of 50 mM AmBic. The reaction was set to 6 μL volume, 45 °C, 60 min with no Reaction Chase. Peptides were eluted in two steps (1 × 25 and 1 × 50 μL) with 50 mM AmBic (no internal cup wash, 10 μL/min). Trypsin (0.5 μg, Promega) was added to the elution and incubated at 37 °C overnight. Biotinylated peptides were eluted with 2 times 15 μL of 10% TFA in acetonitrile (90 μL/min). Syringes were washed with 150 μL of 20% acetonitrile. Biotinylated peptide elution was dried and reconstituted in 50 μL of 50 mM HEPES. The pH was tested and adjusted with sodium hydroxide to pH 6–8.0. Eluate was digested with 0.5 μg of trypsin at 37 °C overnight. Both elutions were cleaned up using Waters Oasis HLB μElution Plate 30 μm (Waters) according to the manufacturer’s instructions.

### Manual Enrichment and Digestion of Biotinylated Proteins

The protocol was used as described by Bartolome et al.^24^ In short, 20 Mio cells were resuspended in 4.75 mL of lysis buffer (see above) and incubated for 1 h at 4 °C. Streptavidin Sepharose High Performance (GE Healthcare) was acetylated by the addition of 10 mM sulfo-NHS acetate (Thermo Fisher Scientific) for 30 min 2 times. For each lysate, 80 μL of equilibrated beads was used. Beads were washed 5 times with 600 μL of 50 mM AmBic. On-bead digest was performed with 200 μL of LysC (5 ng/μL) at 37 °C overnight. The first elution step was achieved using 150 μL of 50 mM AmBic twice. After pooling both fractions, peptides were further digested by adding 1 μg of trypsin and incubating at 37 °C for 3 h. Biotinylated peptides were eluted using 2 times 150 μL of 20% TFA (Biosolve) in acetonitrile (Biosolve). Both fractions were pooled and neutralized to pH 8.0 by adding 50 μL of 200 mM HEPES and sodium hydroxide as necessary. Peptides were digested through the addition of 1 μg of trypsin at 37 °C for 3 h. Both elutions were desalted using Waters Oasis HLB μElution Plate 30 μm (Waters) according to the manufacturer’s instructions.

### Immunoblot

Lysates (10 μg protein) were separated on 4–20% Mini-PROTEAN TGX Precast Protein Gels (Biorad) and blotted onto a Roti-NC transfer membrane (Carl Roth). Proteins were visualized with Ponceau S staining before incubation with 3% BSA (Thermo Fisher) in TBST for 1 h at room temperature. Membranes were then incubated with streptavidin–HRP (1:20,000, Abcam ab7403) for 1 h at room temperature. After washing with TBST, the membranes were incubated with a Pierce ECL Western Blotting Substrate (Thermo Fisher Scientific) and detection was carried out using a ChemiDocTM XRS+ Imaging system (Biorad).

### LC-MS Analysis

For in-depth proteomics analysis, approximately 1 μg of reconstituted peptides was separated using a nanoAcquity ultra-performance liquid chromatography (UPLC) (Waters) coupled online to MS. Peptide mixtures were separated in trap/elute mode, using a trapping (Waters nanoEase M/Z Symmetry C18, 5 μm, 180 μm × 20 mm) and an analytical column (Waters nanoEase M/Z Peptide C18, 1.7 μm, 75 μm × 250 mm). Peptides were eluted via an analytical column with a constant flow of 300 nL/min. During the elution step, the percentage of solvent B increased in a stepwise fashion from 0 to 40% in 90 min. A detailed description of mass spectrometry parameters for DIA analysis can be found in the Supporting Information.

For high-throughput analysis on Evosep, the samples were loaded on Evotips according to the manufacturer’s instructions and more details can be found in the Supporting Information. Peptides were separated using the Evosep One system (Evosep, Odense, Denmark) equipped either with a 8 cm × 150 μm i.d. packed with 1.5 μm Reprosil-Pur C18 beads column (Evosep Performance, EV-1109, PepSep) for the preprogrammed proprietary Evosep gradient of 21 min (60 samples per day, 60SPD) or with a 15 cm × 150 μm i.d. packed with 1.9 μm Reprosil-Pur C18 beads column (Evosep Endurance, EV-1106, PepSep) for the preprogrammed proprietary Evosep gradient of 44 min (30 samples per day, 30SPD). Solvent A was water and 0.1% formic acid, and solvent B was acetonitrile and 0.1% formic acid. LC was coupled to an Orbitrap Exploris 480 instrument (Thermo Fisher Scientific). A detailed description of the MS parameters for DIA analysis can be found in the Supporting Information.

### Data Analysis

DIA raw data were analyzed using the direct DIA pipeline in Spectronaut (Biognosys, v.17, for all experiments, except the experiment to determine substrates with PSMA4 miniTurbo (v.18)). The data were searched against a specific species (*Homo sapiens*, 20,816 entries, release 160,226) and a contaminant (247 entries, release 120,713) Swissprot database. The data were searched with the following variable modifications: Oxidation (M), Acetyl (protein N-term), and Biotin_K. A maximum of 2 missed cleavages for trypsin and 5 variable modifications was allowed. The identifications were filtered to satisfy FDR of 1% on peptide and protein levels. Relative quantification was performed in Spectronaut using the LFQ QUANT 2.0 method with global normalization, precursor filtering percentile using fraction 0.2, and global imputation. Single hit proteins were excluded. The data (candidate table) and data reports (protein quantities) were then exported, and further data analyses and visualization were performed with Rstudio using in-house pipelines and scripts. The significance of the increase of the fold change from proteasome and associates was calculated using the Wilcoxon rank sum test.

To identify ProteasomeID-enriched proteins, we trained a logistic regression binary classifier.^47,48^ A detailed explanation of it can be found in the Supporting Information. Each experiment was analyzed independently.

### Data Availability

Mass spectrometry proteomics data have been deposited to ProteomeXchange Consortium via the MassIVE partner repository, and they are accessible with the identifier MSV000092703 (all BioID data) and MSV000093649 (whole proteome). A detailed step-by-step protocol has been uploaded to www.protocol.io: dx.doi.org/10.17504/protocols.io.kxygxymdwl8j/v2.

## Results

### Implementation of an Automated Workflow for High-Throughput BioID

**Figure 2:**
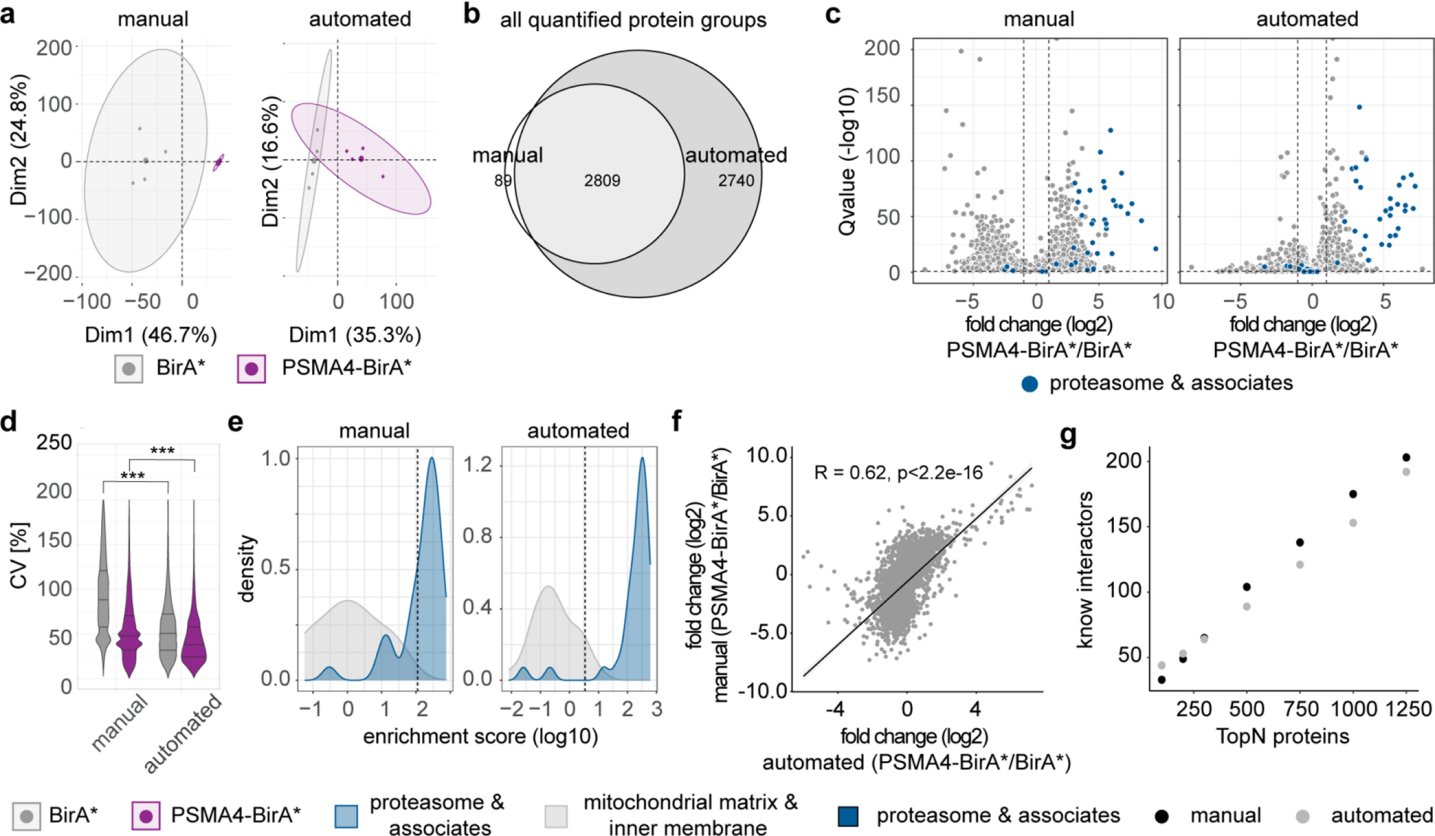
Comparison of manual and optimized automated BioID workflows. A streptavidin pulldown of HEK293 expressing either PSMA-BirA* or BirA* was performed using either the manual or automated workflow. Here, we analyzed the first elution after on bead digest of the pulldowns. Data shown are from four independent expressions of fusion proteins. (a) Principal component analysis of biotin-enriched samples from either the manual or automated workflow. Smaller dots represent replicates, while larger dots are the centroids of each sample group. Ellipses represent 95% confidence intervals. (b) Venn diagram for all quantified protein groups using either the manual (white) or the automated (dark gray) workflow. (c) Volcano plots for all quantified protein groups for both workflows. Proteasome subunits and known associated proteins are highlighted in dark blue. Dashed lines were set at log 2-fold change >1 and *Q*-value <0.05. *n* = 4. (d) Distribution of the coefficient of variation (CV) of both enrichment strategies for BirA* (gray)- and PSMA4-BirA* (purple)-expressing cells. ***p < 0.001, Wilcoxon rank sum test. (e) Distribution of true positive (proteasome subunits and associates, blue) and true negative (mitochondrial matrix and inner membrane proteins, gray) proteins according to their enrichment score. Dashed line marks the calculated cutoff for a false positive rate (FPR) < 0.05. (f) Correlation of the average log 2 ratio of the proteins shared between the workflows. (g) Number of proteins at several topNs that could be identified as either proteasome subunits and associated proteins (dark blue), known proteasome-interacting proteins (PIPs, light turquoise), or the ubiquitin-proteasome system (UPS, dark turquoise) network.

In this study, we used HEK293T cells expressing the PSMA4/alpha3 subunit of the human proteasome tagged with promiscuous biotin ligase BirA* as a proxy for method development. We have previously characterized this cell line both in terms of enrichment efficiency and known associated protein pulldown.^24^ First, we adapted a sample preparation workflow for enrichment and digestion of biotinylated proteins^24^ and implemented it on the liquid handler Bravo AssayMAP (Figure 2a). The workflow has been optimized for parameters, such as lysate concentration, beads pretreatment, and digest time (Figure S1a). Mass spectrometry analysis of peptides obtained by the automated workflow showed clear separation of samples from different experimental groups by principal component analysis (PCA) (Figure 2a) and the expected enrichment of proteasome subunits and associated proteins in the comparison of PSMA4-BirA* vs BirA* control (Figure S1b). Our lab and others have previously shown that modification,^24−27^ here acetylation, of the streptavidin beads prior to loading reduces streptavidin contamination while retaining binding to biotin (Figure S1c). We also tested several washing and priming conditions, as suggested by the manufacturer. We decided to omit the priming step with 1% FA as it caused an increase in poly(ethylene glycol) (PEG) contamination (Figure S1d).^49^ Moreover, washing the columns with a similar bead volume as used with the manual protocol was not sufficient to remove detergents and impacted the enrichment (Figure S1b,d). We achieved successful removal of detergents through increased washing while retaining enrichment of the proteasome and its associated proteins (positive controls)^24^ (Figure S1b,d). Of note, most of the lid components of the 19S proteasome regulatory particle were not enriched (Figure S1b), likely due to an artifact of the sample processing: lysates were frozen before enrichment and this might have led to a partial disassembly of the proteasome.^28^ Most of the lid proteins are in fact not close enough to PSMA4 to achieve direct biotinylation^24^ and were therefore not directly enriched by the streptavidin pulldown. In the subsequent analyses, the lid proteins were omitted from the list of proteasome and associated proteins, as described by Bartolome et al.^24^ (Supporting Table 1), to account for this artifact and to not bias the comparison of different methods.

### Comparison of Manual and Automated BioID Workflows

After optimization of the protocol, we compared our automated workflow with the manual version. All data are derived from four independent experiments from different passages of cells. Almost 95% of the quantified proteins from the manual protocol was shared with the automated workflow (Figure 2b and Supporting Table 1). Most notably, more protein groups were quantified using the automated workflow, including 2740 protein groups that were not identified in the manual version. For both protocols, the proteasome and associated proteins were significantly enriched compared to BirA* control (Figure 2c and Supporting Table 2), but in the manual protocol, we observed a higher number of enriched proteins in BirA* control compared to the automated one. This observation is supported by the fact that the BirA* samples from the manual workflow showed the highest coefficient of variation (CV) for common proteins found under each condition in both workflows (Figure 2d). This might indicate a higher level of unspecific binding of proteins to the matrix as the bead volume is larger in the manual protocol (80 μL beads) than for the automated workflow (5 μL beads). Another difference to be considered is that the beads came from different providers; therefore, they might have slightly different binding properties. As expected, the CVs from the PSMA4-BirA* samples were lower for both workflows. To better assess the signal-to-noise ratio, we analyzed the separation between naturally biotinylated mitochondrial proteins (true negatives) and proteasome subunits and assembly factors (true positives) using a logistic regression classifier that we previously developed.^24^ Briefly, we calculated an “enrichment score”, which combines the negative logarithm of the *Q*-value with the average log 2 ratio, and ranked the proteins according to their enrichment score. We found a clear separation between the true negatives and true positives for both the manual and automated workflow (Figure 2e and Supporting Table 3). In order to compare the performance of the protocols, we used the F1 score, which combines the accuracy of positively identified proteins with the number of retrieved true positives (see the Supporting Information). We found that the overlap between the distributions of enrichment scores for the positive and negative sets was larger for the manual (F1 score: 0.903) than the automated (F1 score: 0.955) protocol, likely due to the higher unspecific binding. Taken together, these observations indicate that the reduced volume of beads in the automated workflow decreased the background binding and, consequently, improved the signal-to-noise ratio.

The fold changes correlated relatively well between the manual and automated workflows (Figure 2f), considering the different matrices of the beads used for enrichment. We then compared how many of the proteins could be identified as proteasome subunits, proteasome-interacting proteins, or are proteins of the ubiquitin-proteasome system.^29−31^ We were able to retrieve similar numbers of known interactors for both workflows at different topNs of the enriched hits obtained by the classifier (Figure 2g).

In summary, our data indicate that the new workflow on the Bravo AssayMAP performs as well as the manual workflow in terms of enrichment of known proteasome subunits and interactors while increasing the number of quantified protein groups. Importantly, the automated workflow enables parallel processing of up to 96 BioID samples in just 2 days, while the normal protocol allows processing of only up to 24 samples at once at the same time.

### Influence of Starting Material and Gradient Length

**Figure 3:**
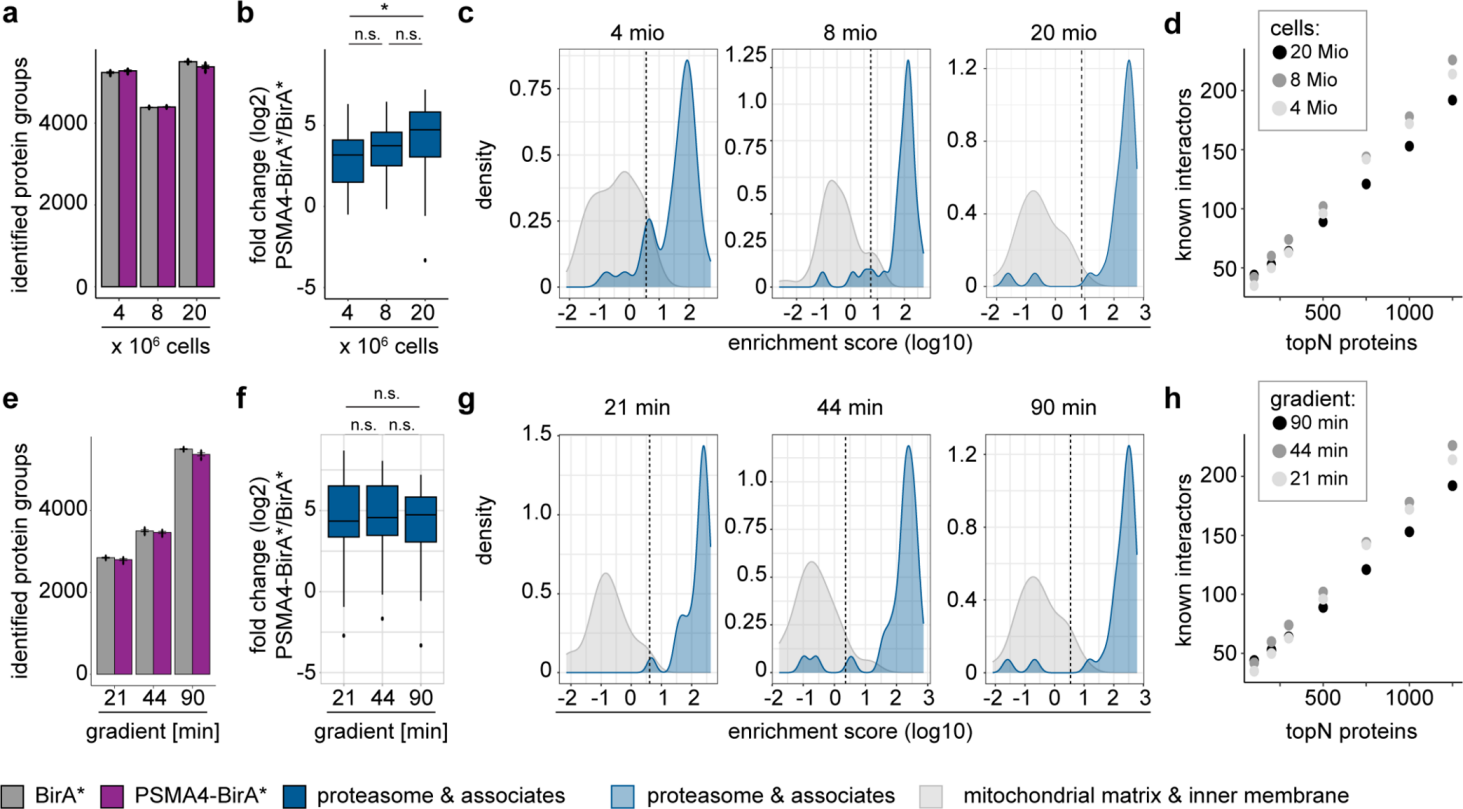
Influence of starting material and gradient length. To test the influence of input and gradient length of LC-MS/MS analysis, we first compared three different cell numbers (4, 8, and 20 Mio) for the automated streptavidin enrichment with the same gradient (90 min gradient). Afterward, we kept the input amount stable while varying the gradient length (21, 44, and 90 min). Only the first elution after on bead digest has been analyzed in this figure. All data shown are from 4 different biological replicates. (a) Number of identified protein groups of proteins separated by cell lines, BirA* (gray) and PSMA4-BirA* (purple) expressing, and input amount. (b) Higher enrichment of proteasome subunits and associates in PSMA4-BirA* samples depending on cell input. *p < 0.05, Wilcoxon rank sum test. (c) Distribution of true positive (blue) and true negative (gray) proteins according to the enrichment score. Dashed line marks the calculated cutoff for a true positive rate (FPR) < 0.05. (d) Number of proteins in several topNs that could be identified as either proteasome subunits and associated proteins (dark blue), known PIPs (light turquoise), or member of the UPS (dark turquoise) network. (e) Number of identified protein groups of proteins separated by cell lines, BirA* and PSMA4-BirA* expressing, and gradient length. (f) Enrichment of proteasome subunits and associates in PSMA4-BirA* samples independent of gradient length. *p < 0.05, Wilcoxon rank sum test. (g) Distribution of true positive and true negative proteins according to the enrichment score. Dashed line marks the calculated cutoff for a true positive rate (FPR) < 0.05. (h) Number of proteins in several topNs that could be identified as either a proteasome subunit and associated proteins, known PIPs or the UPS network.

We reasoned that the higher efficiency and improved signal-to-noise ratio of the automated workflow could enable BioID analysis from lower input material and with faster analysis. Therefore, we used our automated workflow to evaluate the impact of sample input (number of cells used for each enrichment) and gradient length of the LC-MS/MS analysis. We compared 8 combinations of input material and gradient length for a total of 128 MS runs. We found no clear correlation between the amounts of input tested and the identification of protein groups, while we saw a decrease of up to 25% for peptides and precursors in lower input samples (Figures 3a and S2a–c). We observed a significantly higher average log 2-fold change for proteasomal proteins between 20 and 4 Mio cell input (Figures 3b and S2f). This might be explained by the larger total amount of available biotinylated proteasomal proteins with increasing input, which results in more specific binding. Consistently, the separation of true negatives and true positives using our enrichment score was better with increasing cell input, as reflected by F1 scores (4 Mio: 0.861, 8 Mio: 0.870, 20 Mio: 0.955) (Figures 3c and S2e and Supporting Table 3). It is also important to note that the experiments were processed and analyzed independently, and the drop in overall identifications with 8 Mio cells might be due to differences in instrument performances. Despite this, when considering different numbers of topN-enriched proteins, we found no major difference in the recovery of proteasome subunits and associated proteins, previously reported proteasome-interacting proteins (PIPs) and members of the UPS network proteins (Figure 3d). Next, we evaluated the impact of the LC gradient length, keeping cell input fixed at 20 Mio. As expected, gradient length affected the overall identification of precursors, peptides, or protein groups, with more identifications in the longest gradient (Figures 3e and S2a–c). However, it did not influence the relative enrichment of proteasomal and related proteins, as shown by comparison of average log 2 fold changes (Figures 3f and S2f), and there was no major difference in the separation between enrichment scores of true negatives and true positives (F1 scores for 21 min: 0.923, 44 min: 0.938, 90 min: 0.955) (Figures 3g and S2e and Supporting Table 3). Despite the decrease of data-points-per-peak (DPPP) with shorter gradients (6 DPPP for 90 min gradient, 3.5 DPPP for 21 min gradient), the quantitation was not affected, as shown by the stability of CVs across different gradients (Figure S2d).

Interestingly, we observed a similar recovery of proteasome-related proteins among the topN-enriched proteins using shorter gradients (Figure 3h). From these results, we conclude that shorter gradients down to 21 min can be used in combination with our automated enrichment workflow, enabling higher throughput, while still allowing the identification of the most abundant and significant interaction candidates.

### Automated Workflow to Increase Identification of Biotinylation Sites

**Figure 4:**
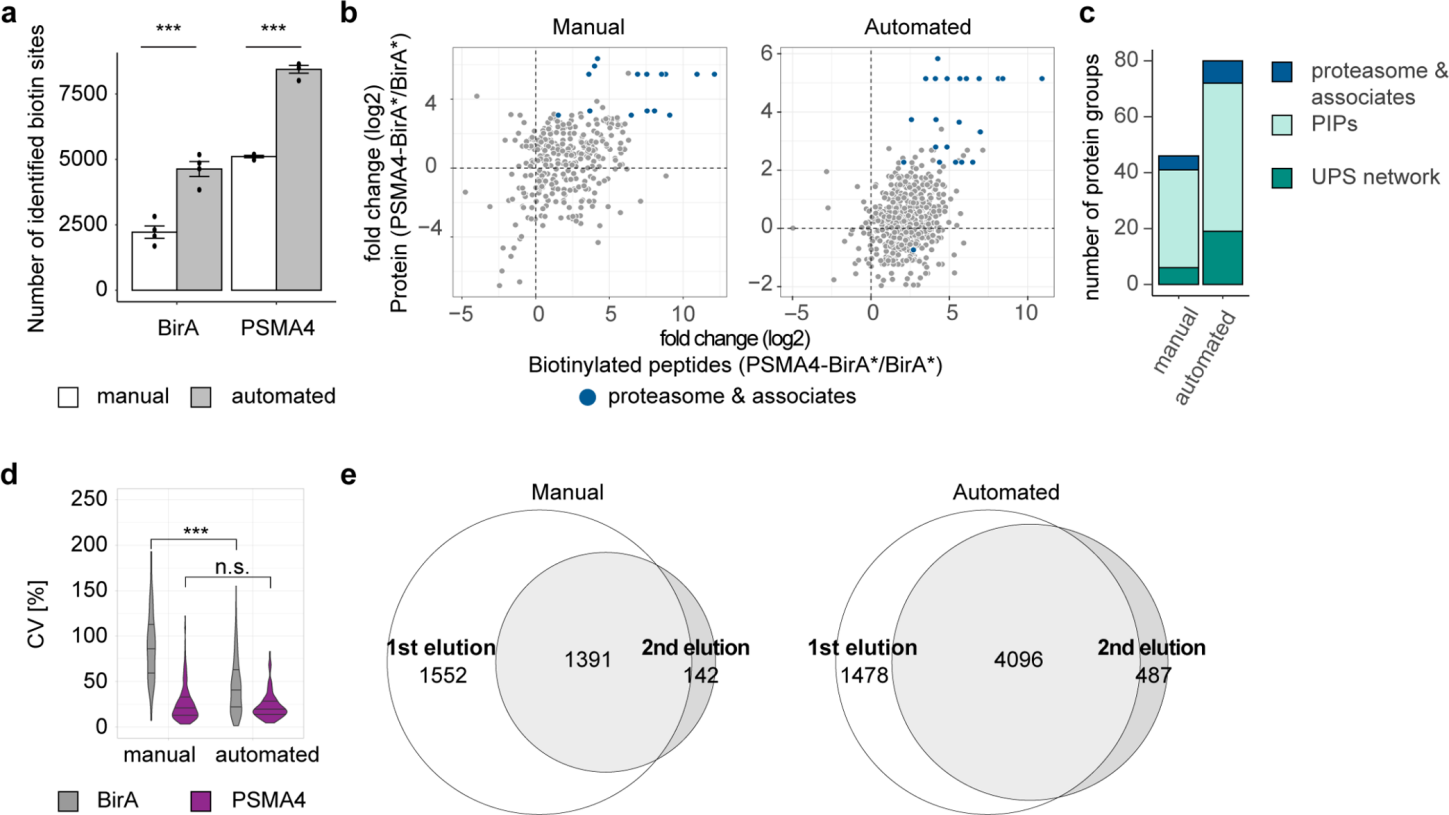
Optimized automated workflow increases the identification of biotinylation sites. A streptavidin pulldown of HEK293 expressing either PSMA-BirA* or BirA* was performed using either the manual or automated workflow. Here, we analyzed the acidic second elution of the pulldowns containing most of the biotinylated peptides. Data shown are from four different biological replicates. (a) Significantly more biotin sites identified using the automated (dark gray) compared to the manual (white) workflow (one-way ANOVA, *p*-value: BirA = 6.36 × 10^–4^, PSMA = 6.28 × 10^–7^). (b) Comparison of protein-level average log 2-fold enrichments based on the first elution after on-bead digestion and the biotinylated peptide-level average log 2-fold enrichments from the acidic second elution. Only biotinylated peptides derived from proteins identified in both elutions were used. Proteasome subunits and known associated proteins are highlighted in dark blue. (c) Number of identified biotinylated proteins of the proteasome and associates, PIPs or the UPS network in the optimized automated vs manual workflow. (d) Distribution of the coefficient of variation (CV) of both enrichment strategies for BirA* (gray) and PSMA4-BirA* (purple) expressing cells for biotinylated protein groups. ***p < 0.001, Wilcoxon rank sum test. (e) Venn diagram for all quantified protein groups identified in either the first elution (white) or the acidic second elution (dark gray) using either the manual or automated workflow.

Finally, we evaluated the enrichment of biotinylated peptides using a highly acidic second elution step from 20 Mio cells input analyzed by using a 90 min gradient LC-MS/MS setup. Using direct DIA analysis, we could show that the automated workflow enabled the detection of significantly more biotinylated sites in comparison to its manual counterpart (Figure 4a). As expected, the log 2-fold changes obtained for the biotinylated peptides correlated with the ones obtained for the corresponding proteins from the on-bead digestion fraction, with proteasome subunits and related proteins displaying the strongest enrichment (Figure 4b and Supporting Table 4). More biotinylated proteins from the automated (80 protein groups) compared to the manual (44 protein groups) workflow were assigned to known interactors (proteasome and associates, PIPs or the UPS network) (Figure 4c). The fraction of identified known interactors in the automated pipeline was comparable between proteins assigned using protein abundance (14.5%: 262 interactors of 1803 proteins with classifier FPR < 0.05 with 42 in the 100 most significant proteins) and for biotinylated proteins (17.8%: 77 interactors of 432 proteins with at least one significantly enriched biotinylated peptide with 34 in the 100 most significant proteins). The CVs for common proteins between both workflows in the second elution step were higher for BirA samples with the manual workflow, showing significantly higher CVs compared to the automated workflow (Figure 4d). For both workflows, the CVs from PSMA4-BirA* samples were significantly lower and comparable between methods. Most proteins quantified from the acidic second elution step were also detected in the first elution step (Figure 4e).

These results show that the optimized automated workflow improves the detection of biotinylated peptides and therefore the identification of direct interactors.

### Application of Optimized Automated BioID to Improve the Detection of Proteasome Substrates

**Figure 5:**
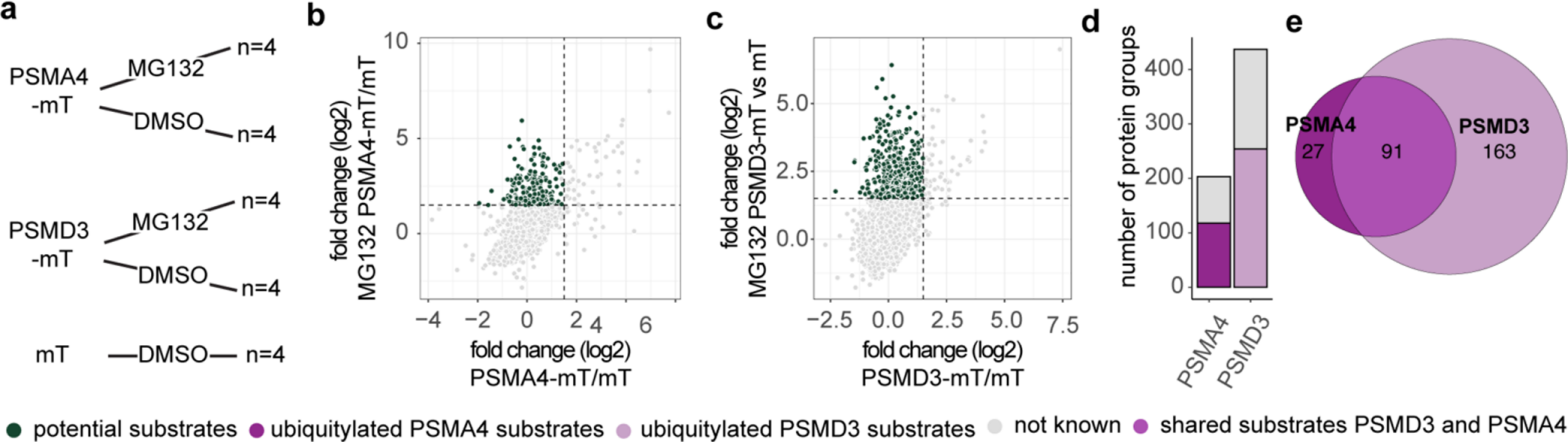
Tagging different proteasome subunits to identify proteasome substrates. (a) Workflow to identify the best construct for substrate detection. Two proteasome subunits from either the 19S (PSMD3) or 20S (PSMA4) complex were fused to miniTurbo. To enhance detection of substrates, the proteasome inhibitor MG132 was added. Data shown here are from different biological replicates. Differential expression of (b) PSMA4 miniTurbo or (c) miniTurbo-PSMD3 compared to miniTurbo with MG132, or vehicle control was plotted against each other. Proteins enriched after inhibition (fold change (log 2) > 1.5, *Q*-value <0.05), but not with the vehicle control (fold change (log 2) < 1.5, *Q*-value <0.05) were defined as potential substrates (dark green). (d) Number of potential substrates that have been shown by Trullson et al. to increase ubiquitylation after MG132 treatment. (e) Venn diagram comparing ubiquitylated protein groups identified using PSMA4 miniTurbo or miniTurbo-PSMD3.

Next, we wanted to demonstrate the application of the optimized workflow for improving the detection of proteasome substrates by proximity-dependent labeling, an approach that we have previously developed using the manual BioID workflow.^24^ Therefore, we tagged two subunits of the proteasome with miniTurbo, as it has been shown to biotinylate more rapidly.^32^ Furthermore, the proteasome was inhibited with MG132 to prolong the duration of the interaction between the proteasome and its substrates and reduce nontryptic peptide generation, as previously shown.^24^ To test whether a subunit from the 19S would improve the detection of substrates, PSMD3 was chosen in addition to the previously characterized 20S subunit PSMA4 (Figures 5a and S3a,b). Proteins that were found enriched from lysates of cells expressing PSMA4 miniTurbo or miniTurbo-PSMD3 treated with MG132 but not in samples treated with DMSO were defined as potential substrates (Figure 5b–c and Supporting Table 5). To exclude any influence on the proteome of the cells from the MG132 treatment, we checked for upregulated proteins in the whole cell analysis that could end up being defined as potential substrates in the BioID experiment: we found that proteome regulation had a negligible influence (2% of the significantly upregulated proteins in our BioID, Figure S3c). While we detected 206 potential substrates with PSMA4 miniTurbo, almost double the number of potential substrates (437) was identified with miniTurbo-PSMD3 (Figure 5d). Out of these, more than half have been previously reported to display an increased ubiquitylation in response to proteasome inhibition.^33^ Most of the known substrates found with PSMA4 miniTurbo were also found with miniTurbo-PSMD3 (Figure 5e). However, the majority of these known substrates from our miniTurbo-PSMD3 analysis were found as significantly enriched exclusively with this construct. Taken together, these results suggest that the miniTurbo-PSMD3 construct is better for detection of proteasome substrates by proximity-dependent labeling than PSMA4 miniTurbo.

## Discussion

In recent years, proximity-dependent biotinylation has become the method of election to study protein–protein interactions and it has been vastly developed and optimized for many different applications, including studies of protein complexes and cell compartmentalization.^5,9,34^ The biotinylation reaction has been improved by using different promoters or antibody-based delivery of the biotin ligase.^35−37^ Some recent developments include the combination of mass spectrometry for posttranslational modifications (PTMs) with proximity labeling methods to elucidate the role of PTMs on the localization of proteins.^7,8^

The increasing complexity of experimental designs for this type of approach, which often requires multiple controls to enable correct interpretation of the results, motivated us to develop a robust and higher throughput workflow for sample preparation and MS analysis. By combining automated sample preparation on a robot with short gradient LC-MS methods, our pipeline reduces the sample processing and measuring time up to four times compared to the manual protocol and enables analysis from the reduced input material down to one-fifth of the usual pipeline.

The Agilent Bravo^38^ has been used to perform automated BioID experiments. To our knowledge, only one previous publication^39^ described the systematic development of an optimized automated workflow for proximity-dependent labeling on an alternative platform. While the previous manuscript optimized parameters for the DIA acquisition, we focused mainly on increasing the throughput by minimizing the analysis time (gradient length) and reducing the amount of input material required. With the cell line and bait protein used in our study, we have shown that satisfactory results can be obtained with as little as 4 Mio cells input. We did not test lower numbers of cells. Proximity-dependent labeling has been successfully applied in organisms to study protein–protein interactions in vivo^3,40,41^ and recently has been used to study cell-type specific proteomes in the brain.^42^ Protein complexes like the proteasome can have different compositions dependent on the cell type,^43^ and studying these complexes could give valuable insight into their cell-type specific functions. The lower sample input required by our optimized automated BioID workflow could enable this type of study also in less abundant cell types. However, this would need to be tested for each specific application since other factors, such as the expression level of the bait protein, might influence the yield of the streptavidin enrichment.

Finally, we also improved the detection of biotinylated peptides. The detection of direct biotinylation typically indicates that the protein was in close proximity to the bait, and therefore, it likely represents a direct interaction partner. This is important as, during the streptavidin pulldown, not only direct interactors but also their binding partners can be enriched. Several attempts have been made to enhance the detection of these biotinylated proteins by modifying biotin affinity reagents,^16,19^ performing pulldown on peptides (DiDBiT)^20^ or a combination of both.^13^ We adapted the protocol developed by Bartolome et al.,^24^ which uses a very mild washing buffer with an optimized on bead digest to ensure capturing indirect interactors and combines it with a second highly acidic harsher eluting step to identify direct interactors by detecting the biotinylation sites. Because of the harsher buffer needed to break the streptavidin–biotin bond, the acidic second elution step is especially sensitive to variations in sample handling, e.g., contact time between the elution buffer and streptavidin beads. For example, excessively long elution times can lead to denaturation of streptavidin and release of its monomers, which could negatively influence the downstream LC-MS analysis. By implementing the workflow on a liquid handler, we reduced the variability of this step and enabled a more reproducible and deeper quantification of biotinylated peptides.

The characteristics highlighted above make our workflow suitable for most proximity labeling experiments. Furthermore, our method could be easily adapted to other protocols that rely on the enrichment of biotinylated peptides, e.g., surface proteomics or protein synthesis analysis by incorporation of amino acid analogues that can be biotinylated via click chemistry.^44−46^

## Supporting Information Available

Extended experimental section on LC-MS/MS setup and data analysis; setting up the automated workflow (Figure S1); influence of input amount and gradient length of the LC-MS/MS analysis on interactor identification (Figure S2); tagging different proteasome subunits to identify proteasome substrates (Figure S3), and entire membrane of Western blot analysis (PDF)
List of proteasome and associated proteins and list of proteins identified either through the manual or automated workflow (Table S1) (XLSX)
Data on enrichment with either the manual or automated workflow for PSMA4-BirA* and overlap with previous studies (Table S2) (XLSX)
Protein groups enriched for all PSMA4-BirA* data set by the classifier algorithm (Table S3) (XLSX)
Biotinylation sites enriched with PSMA4-BirA* using the manual or automated workflow (Table S4) (XLSX)
Data on enrichment with PSMA4 miniTurbo or miniTurbo-PSMD3 and overlap with ubiquitilation sites that have been reported to increase after MG132 inhibition (Table S5) (XLSX)
