# Supplementary material for: Optimized Automated Workflow for BioID Improves Reproducibility and Identification of Protein–Protein Interactions
Source: J Proteome Res. 2024 Sep 4;23(10):4359–68. doi: 10.1021/acs.jproteome.4c00308 (PMC11460324; doi:10.1021/acs.jproteome.4c00308)
Supplement: Supplementary file 1 — pr4c00308_si_001.pdf [file pr4c00308_si_001.pdf]

Automated workflow for BioID improves reproducibility and identification of protein-protein interactions

Emilio Cirri[1], Hannah Knaudt[1], Domenico Di Fraia[1], Nadine Pömpner[1], Norman Rahnis[1], Ivonne Heinze[1], Alessandro Ori[1,#], Therese Dau[1,#]

1 - Leibniz Institute on Aging - Fritz Lipmann Institute (FLI), Jena, Germany

# correspondence to: alessandro.ori@leibniz-fli.de and therese.dau@leibniz-fli.de

Keywords: BioID, proximity labelling, mass spectrometry, automation, high throughput.

Supplemental Information

Contents:

Extended experimental section on LC-MS/MS setup and data analysis

Figure S1: Setting up the automated workflow.

Figure S2: Influence of input amount and gradient length of the LC-MS/MS analysis on interactors identification.

Figure S3: Tagging different proteasome subunits to identify proteasome substrates.

Supplementary table 1: List of proteasome and associated proteins, and list of proteins identified either through the manual or automated workflow.

Supplementary table 2: Data on enrichment with either the manual or automated workflow for PSMA4-BirA* and overlap with previous studies

Supplementary table 3: Protein groups enriched for all PSMA4-BirA* dataset by the classifier algorithm

Supplementary table 4: Biotinylation sites enriched with PSMA4-BirA* using the manual or automated workflow.

Supplementary table 5: Data on enrichment with PSMA4-miniTurbo or miniTurbo-PSMD3 and overlap with ubiquitilation sites that have been reported to increase after MG132 inhibition.

Supplementary data: entire membrane of western blot analysis

Supplementary experimental section

LC-MS Data independent acquisition (DIA) methods

For in-depth proteomics analysis, approximately 1µg of reconstituted peptides were separated using a nanoAcquity UPLC (Waters) coupled online to the MS. Peptide mixtures were separated in trap/elute mode, using a trapping (Waters nanoEase M/Z Symmetry C18, 5µm, 180 µm x 20 mm) and an analytical column (Waters nanoEase M/Z Peptide C18, 1.7µm, 75µm x 250mm). The outlet of the analytical column was coupled directly to an Orbitrap Fusion Lumos mass spectrometer (Thermo Fisher Scientific) using the Proxeon nanospray source. Solvent A was water, 0.1% formic acid and solvent B was acetonitrile, 0.1% formic acid. The samples were loaded with a constant flow of solvent A, at 5 µL/min onto the trapping column. Trapping time was 6 min. Peptides were eluted via the analytical column with a constant flow of 300 nL/min. During the elution step, the percentage of solvent B increased in a nonlinear fashion from 0% to 40% in 90 min. Total runtime was 115 min, including cleanup and column re-equilibration. The peptides were introduced into the mass spectrometer via a Pico-Tip Emitter 360 µm OD x 20 µm ID; 10 µm tip (New Objective) and a spray voltage of 2.2 kV was applied. The capillary temperature was set at 300 °C. The RF lens was set to 30%. Full scan MS spectra with mass range 350-1650 m/z were acquired in profile mode in the Orbitrap with resolution of 120,000 FWHM. The filling time was set at maximum of 20 ms with an AGC target of 5×10^5 ions. DIA scans were acquired with 34 mass window segments of differing widths across the MS1 mass range. The HCD collision energy was set to 30%. MS/MS scan resolution in the Orbitrap was set to 30,000 FWHM with a fixed first mass of 200m/z after accumulation of 1×10^6 ions or after filling time of 70ms (whichever occurred first). Data were acquired in profile mode. For data acquisition and processing Tune version 3.5 and Xcalibur 4.5 were employed.

For high-throughput analysis on Evosep, the samples were loaded on Evotips according to the manufacturer's instructions. In short, Evotips were first washed with Evosep buffer B (0.1% formic acid in acetonitrile), conditioned with 100% isopropanol and equilibrated with Evosep buffer A (0.1% acetonitrile). Afterwards samples were loaded on the Evotips and washed with Evosep buffer A. The loaded Evotips were topped up with buffer A and stored until the measurement. Peptides were separated using the Evosep One system (Evosep, Odense, Denmark) equipped either with a 8 cm x 150 µm i.d. packed with 1.5 µm Reprosil-Pur C18 beads column (Evosep Performance, EV-1109, PepSep) for the pre-programmed proprietary Evosep gradient of 21 min (60 samples per day, 60SPD), or with a 15 cm x 150 µm i.d. packed with 1.9 µm Reprosil-Pur C18 beads column (Evosep Endurance, EV-1106, PepSep) for the re-programmed proprietary Evosep gradient of 44 min (30 samples per day, 30SPD). Solvent A was water and 0.1% formic acid and solvent B was acetonitrile and 0.1% formic acid. The LC was coupled to an Orbitrap Exploris 480 (Thermo Fisher Scientific) using the Proxeon nanospray source. The peptides were introduced into the mass spectrometer via a PepSep Emitter 360-µm outer diameter ×

20-µm inner diameter, heated at 300 °C, and a spray voltage of 2 kV was applied. The radio frequency ion funnel was set to 30%.

For DIA data acquisition of the 60SPD method, full scan mass spectrometry (MS) spectra with mass range 350–1650 m/z were acquired in profile mode in the Orbitrap with resolution of 120,000 FWHM. The default charge state was set to 2+. The filling time was set at a maximum of 45 ms (60SPD) or 60 ms (30SPD) with a limitation of 3×10^6 ions. DIA scans were acquired with 35 (60SPD) or 40 (30SPD) mass window segments of differing widths across the MS1 mass range. Higher collisional dissociation fragmentation of 30%) was applied and MS/MS spectra were acquired with a resolution of 15,000 FWHM (60SPD) or 30,000 FWHM (30SPD) with a fixed first mass of 200 m/z after accumulation of 1×10^6 ions or after filling time of 37 ms (whichever occurred first). Data were acquired in profile mode.

For DIA data acquisition of the 30SPD method, full scan mass spectrometry (MS) spectra were acquired as above, with the only difference of the filling time set at at a maximum of 60 ms with a limitation of 3 × 106 ions. DIA scans were acquired with 40 mass window segments of differing widths across the MS1 mass range. Higher collisional dissociation fragmentation of 30% was applied and MS/MS spectra were acquired with a resolution of 30,000 FWHM with a fixed first mass of 200 m/z after accumulation of 1 × 106 ions or after filling time of 45 ms (whichever occurred first). Data were acquired in profile mode. For data acquisition and processing of the raw data Xcalibur 4.5 (Thermo) and Tune version 4.0 were used.

Binary classifier for ProteasomeID enriched proteins

The classifier was trained using known proteasome interactors as positive class, and mitochondrial matrix proteins as negative. Mitochondrial matrix proteins are naturally biotinylated, but they are not expected to interact directly with the proteasome under homeostatic conditions. To distinguish between these two classes, we performed prediction using an enrichment score derived from multiplying the average log2 ratio and the negative logarithm of the q-value obtained from a differential protein abundance analysis performed against the BirA* control line. Before analysis, any missing data points were removed from the dataset. To assess the performance of the binary classifier, and optimize its parameters, a 10-fold cross-validation approach was adopted. The dataset was randomly partitioned into ten subsets, with nine subsets used for training and one subset for validation in each iteration. This process was repeated thirty times, and results were averaged using the mean value to ensure the robustness of the results. Logistic regression was employed as the classification method using the caret package in R [47]. To determine an optimal threshold for classification, the false positive rate (FPR) was set at 0.05. The threshold yielding an FPR closest to the target value of 0.05 was selected as the final classification threshold. Following model training and threshold selection, the classifier was applied to predict the class labels of additional proteins not used in the training process. The enrichment score and class labels for the new data were provided as input to the trained model. All statistical analyses were performed using R version 4.1.3. The pROC [48] and caret [47] packages were employed for ROC analysis and logistic regression, respectively. The F1 score used to compare conditions was calculated as the

harmonic mean of precision and recall: TP/(2TP+FP+FN), TP: true positive, FP: false positive, FN: false negative.

Extraction of PEG contaminations

To extract and quantify PEG contamination, the Skyline contamination template developed by Jardin[49].

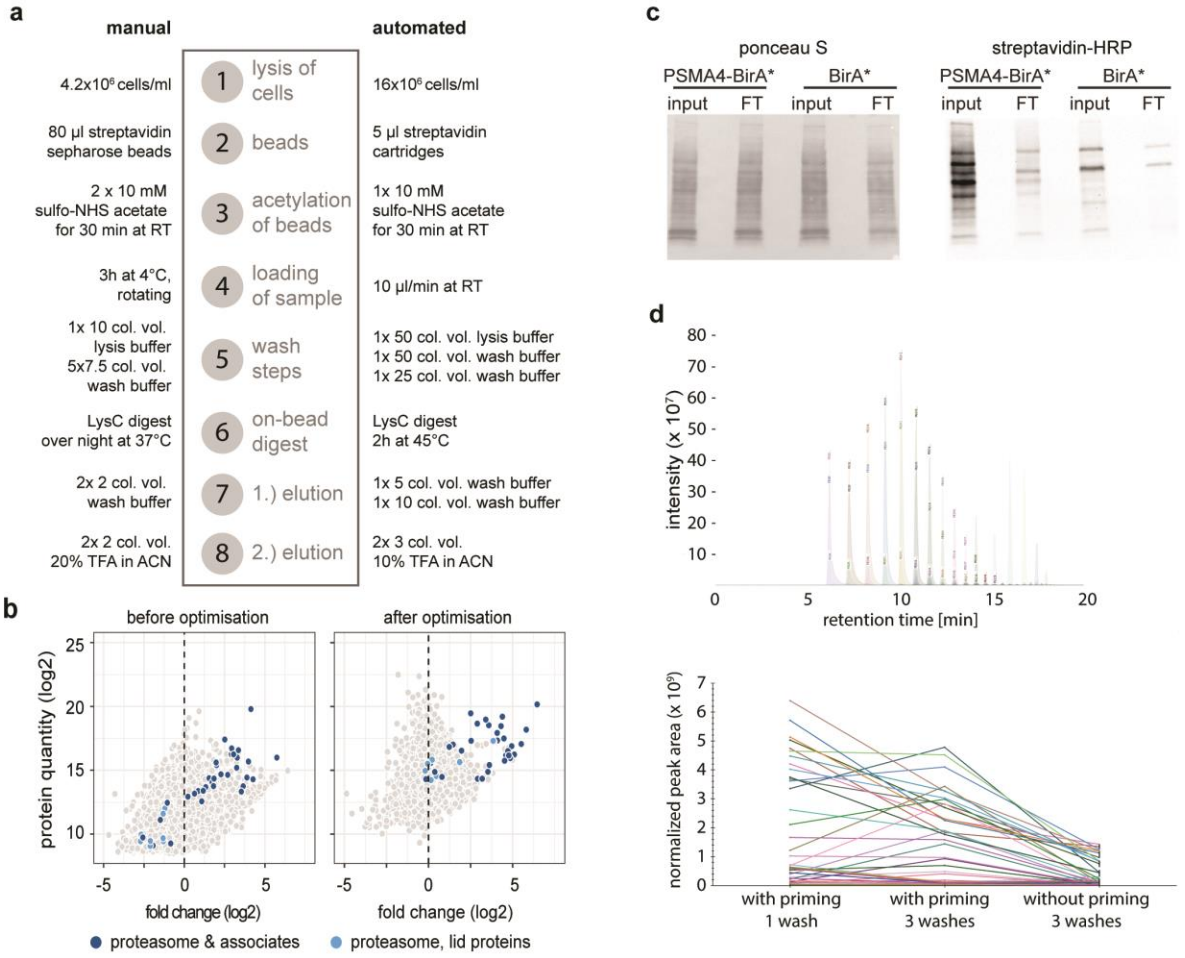

Figure S1: Setting up the optimized automated workflow. (a) A schematic overview comparing the manual and automated workflow after optimisation (b) MA plot of enriched proteins with proteasomal proteins highlighted in dark blue and proteasome lid proteins in light blue before and after washing steps were tested. (c) Input and flowthrough (FT) were analysed by SDS-PAGE and western blotting using streptavidin-HRP (right panel). Ponceau S was used as a loading control (left panel). HRP: horseradish peroxidase. (d) Example of an extracted chromatogram of PEG peaks (upper panel) with corresponding

extracted and normalized peak areas (lower panel) when (1) priming beads with 0.1 % FA and one simple wash step, (2) priming the beads and increasing the wash steps to three, or (3) washing the beads three times without priming. Graphs are directly exported from Skyline and each color corresponds to a precursor ion.

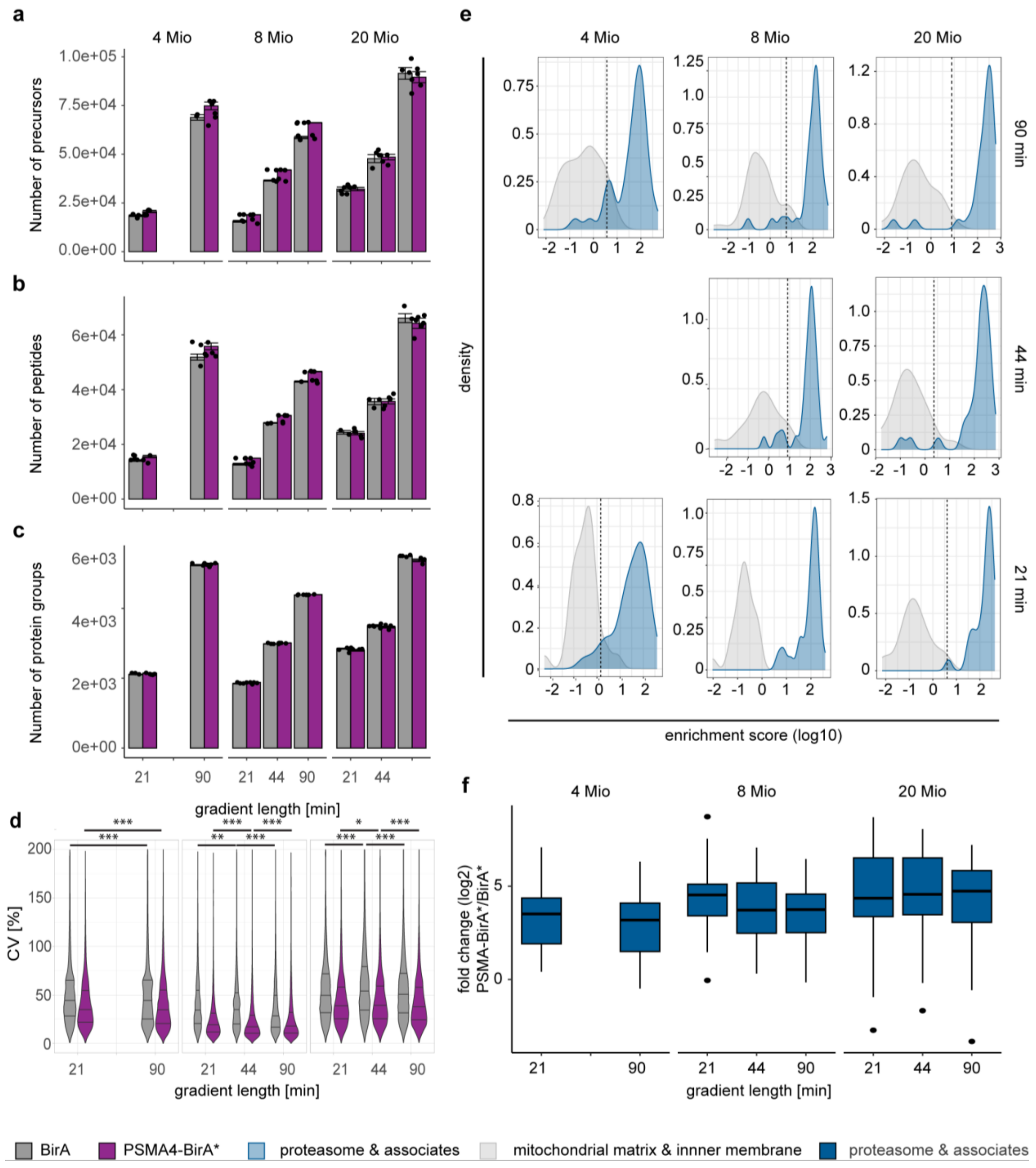

Figure S2: Influence of input amount and gradient length of the LC-MS/MS analysis on interactors identification. All experiments depicted in this figure show results depending on input amount (4, 8 and 20 Mio cells) and gradient length (21, 44 and 90 min). Data shown are derived from 4 independent experiments. The identification of (a) precursors, (b) peptides and (c) protein groups was impacted by the gradient, but not the cell input. For 4 and 8 Mio cells, less precursors, peptides and proteins were identified in BirA* compared to PSMA-BirA* samples. (d) Violin plot showing the distribution of CVs in BirA* and PSMA4-BirA* cell lines. (e) Distribution of true positive (blue) and true negative (gray) proteins according to the enrichment score. Dashed line marks the cutoff for a true positive rate (FPR) < 0.05. (f) Enrichment of proteasome subunits and associates in PSMA4-BirA* samples.

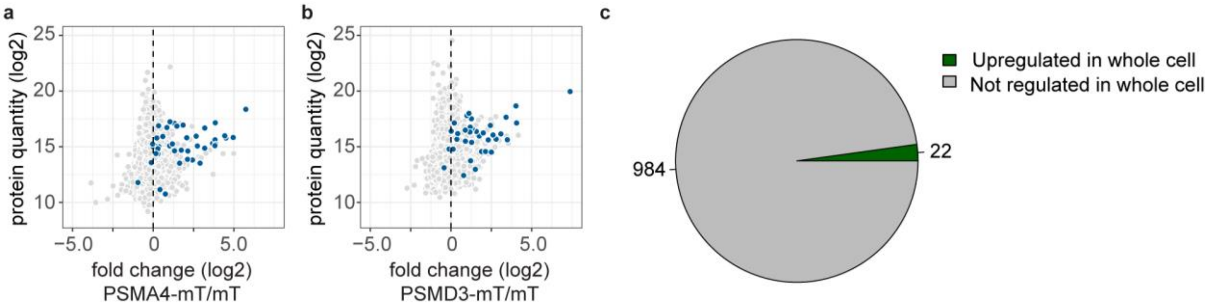

Figure S3: Tagging different proteasome subunits to identify proteasome substrates. Two proteasome subunits from either the 19S (PSMD3) or 20S (PSMA4) complex were fused to miniTurbo. Data shown here are from 4 independent experiments. MA plot of enriched proteins with proteasomal proteins highlighted in dark blue from either (a) PSMA4-miniTurbo or (b) PSMD3-mini-Turbo expressing cell lines. (c) Absolute number of potential proteasome substrates and interactors that were regulated in the whole proteome.
